# Ambient Carbon Monoxide and Fine Particulate Matter in Relation to Preeclampsia and Preterm Delivery in Western Washington State

**DOI:** 10.1289/ehp.1002947

**Published:** 2011-01-24

**Authors:** Carole B. Rudra, Michelle A. Williams, Lianne Sheppard, Jane Q. Koenig, Melissa A. Schiff

**Affiliations:** 1 Department of Social and Preventive Medicine, School of Public Health and Health Professions, State University of New York, Buffalo, New York, USA; 2 Center for Perinatal Studies, Swedish Medical Center, Seattle, Washington, USA; 3 Department of Epidemiology; 4 Department of Biostatistics and; 5 Department of Environmental and Occupational Health Sciences, School of Public Health, Seattle, Washington, USA; 6 Harborview Injury Prevention and Research Center, Harborview Medical Center, Seattle, Washington, USA

**Keywords:** air pollution, carbon monoxide, fine particulate matter, preeclampsia, pregnancy preterm delivery

## Abstract

**Background:**

Preterm delivery and preeclampsia are common adverse pregnancy outcomes that have been inconsistently associated with ambient air pollutant exposures.

**Objectives:**

We aimed to prospectively examine relations between exposures to ambient carbon monoxide (CO) and fine particulate matter [≤ 2.5 μm in aerodynamic diameter (PM_2.5_)] and risks of preeclampsia and preterm delivery.

**Methods:**

We used data from 3,509 western Washington women who delivered infants between 1996 and 2006. We predicted ambient CO and PM_2.5_ exposures using regression models based on regional air pollutant monitoring data. Models contained predictor terms for year, month, weather, and land use characteristics. We evaluated several exposure windows, including prepregnancy, early pregnancy, the first two trimesters, the last month, and the last 3 months of pregnancy. Outcomes were identified using abstracted maternal medical record data. Covariate information was obtained from maternal interviews.

**Results:**

Predicted periconceptional CO exposure was significantly associated with preeclampsia after adjustment for maternal characteristics and season of conception [adjusted odds ratio (OR) per 0.1 ppm = 1.07; 95% confidence interval (CI), 1.02–1.13]. However, further adjustment for year of conception essentially nullified the association (adjusted OR = 0.98; 95% CI, 0.91–1.06). Associations between PM_2.5_ and preeclampsia were nonsignificant and weaker than associations estimated for CO, and neither air pollutant was strongly associated with preterm delivery. Patterns were similar across all exposure windows.

**Conclusions:**

Because both CO concentrations and preeclampsia incidence declined during the study period, secular changes in another preeclampsia risk factor may explain the association observed here. We saw little evidence of other associations with preeclampsia or preterm delivery in this setting.

Preeclampsia is a disorder that affects about 5–8% of pregnant American women [American College of Obstetricians and Gynecologists (ACOG) 2002]. It is diagnosed by the onset of hypertension and proteinuria in mid-pregnancy and is characterized by systemic endothelial dysfunction, vasoconstriction, and reduced organ perfusion (Roberts et al. 2003). It is a major cause of intrauterine growth restriction, low birth weight, and maternal death (ACOG 2002; Roberts et al. 2003). Preeclampsia shares many risk factors with cardiovascular disease and is itself a risk factor for future cardiovascular morbidity and mortality (Kaaja and Greer 2005; Newstead et al. 2007). It can also lead to preterm delivery, a cause of infant mortality, and numerous adverse outcomes, including acute respiratory, gastrointestinal, and immunologic problems and longer-term motor, cognitive, and growth problems (Institute of Medicine 2007). The strength of association between preeclampsia and preterm delivery differs according to such characteristics as gestational age at preterm delivery and the timing of membrane rupture in relation to onset of labor (Ananth et al. 1997). Preterm deliveries occurred in 12.3% of U.S. live births in 2008, an increase of approximately 20% since 1990 (Hamilton et al. 2006).

Ambient air pollutants have been shown to elevate risks of cardiovascular and respiratory diseases (Brook 2008; Eder et al. 2009). They may also increase the risks of preeclampsia and preterm delivery via several pathways, including placental and fetal hypoxia, inflammation, and oxidative stress (Kannan et al. 2006; Roberts et al. 2003). Several investigations have shown mixed evidence of associations between preterm delivery and ambient air pollutant exposures typical of U.S. cities (Brauer et al. 2008; Darrow et al. 2009; Liu et al. 2003; Parker et al. 2008; Ritz et al. 2007; Wilhelm and Ritz 2005; Wu et al. 2009; Zeka et al. 2008). Preeclampsia has also been associated with air pollution, although it has been studied less often than preterm delivery (Rudra and Williams 2006; Woodruff et al. 2008; Wu et al. 2009).

We aimed to examine exposures to two ambient air pollutants before and during pregnancy in relation to risks of preeclampsia and preterm delivery. We developed exposure models to predict individual exposures to ambient carbon monoxide (CO) and fine particulate matter [≤ 2.5 μm in aerodynamic diameter (PM_2.5_)]. We used data from a well-characterized cohort of pregnant women living in the Puget Sound airshed during a time of fairly stable PM_2.5_ concentrations and substantial CO decline [Puget Sound Clean Air Agency (PSCAA) 2007]. At the International Conference on Environmental Epidemiology and Exposure in 2006, we presented results of an analysis of air pollutant exposures and preeclampsia risk in the subset of participants who enrolled between 1996 and 2002 (Rudra and Williams 2006). In the present analysis, we report preeclampsia findings based on the full cohort and also examine preterm delivery.

## Methods

### Study population

We conducted a prospective analysis using data from the Omega Study, a pregnancy cohort study previously described in detail (Butler et al. 2004). Participants were women attending prenatal care clinics affiliated with Swedish Medical Center in Seattle and Tacoma General Hospital in Tacoma, Washington. Eligible women initiated prenatal care before 20 weeks of gestation. Women were ineligible if they were < 18 years of age, were non-English speakers, or did not plan to deliver at either hospital. Participants completed an interviewer-administered questionnaire soon after enrollment (mean ± SD gestational age, 15.9 ± 4.8 weeks). This questionnaire was used to collect information on sociodemographic, anthropometric, behavioral, medical, and reproductive characteristics. After delivery, study personnel abstracted information on the pregnancy course and outcome from medical records. Study procedures were approved by the institutional review boards of both hospitals. All participants provided written informed consent.

We used data from women recruited between 1996 and 2006. During this period, 4,000 (79%) of 5,063 invited women enrolled in the study. Of these, 79 experienced early pregnancy losses, 68 moved or delivered elsewhere, and 169 were lost to follow-up due to unknown delivery outcome or a missing medical record. Of the 3,675 women who completed the study, we included 3,509 (95%) in this analysis. We excluded 145 participants for whom we were unable to accurately assess exposures: 121 had nongeocodable residential addresses, and 24 lived outside the study area of King, Kitsap, Pierce, and Snohomish counties. These participants were less likely, on average, to be nulliparous than women who remained in the analytic sample (51% vs. 60%) but were otherwise similar. We also excluded 21 additional women who were missing information on one or more covariates chosen *a priori* for final models, as described below. Distributions of air pollutant exposures did not substantially differ between women with missing and nonmissing covariate information.

### Air pollutant exposure estimation

We predicted monthly ambient CO and PM_2.5_ exposures using two multivariable linear regression models that included terms for environmental characteristics, month, and year. We did not examine other criteria pollutants (ozone, nitrogen oxides, sulfur dioxide, and lead) because there were too few monitoring sites to construct exposure models (PSCAA 2007). The CO model has been previously described in detail (Rudra et al. 2010b). Our models were constructed using CO and PM_2.5_ measurements collected daily between 1996 and 2006 at monitoring sites administered by the PSCAA. The sites were located by the PSCAA using U.S. Environmental Protection Agency (EPA) criteria to ensure a consistent and representative measure of air quality (PSCAA 2007). We collapsed daily measurements into monthly average concentrations, resulting in 890 CO concentrations from 15 sites and 803 PM_2.5_ concentrations from 12 sites. For more site characteristics and a map of monitoring sites and participants’ residences, see Supplemental Material, Table 1, Figure 1 (doi:10.1289/ehp.1002947).

### Air pollutant predictor variables

We evaluated several characteristics as potential predictors of monthly average pollutant concentrations at the monitoring sites. We mapped and measured characteristics at each site using ArcMap (version 9.2; ESRI, Redlands, CA). We used 2001 traffic count data from the Washington Department of Transportation to estimate annual average traffic volume on the nearest major road (federal or state highway) within circular buffers with radii of 250 and 500 m (Washington State Department of Transportation 2002). We also measured distance to the nearest major road. We used Census 2000 TIGER line files to estimate street density (kilometers per square kilometer) within 100-, 250-, 500-, and 1,000-m buffers (U.S. Census Bureau 2002). As has been done in previous literature, we chose *a priori* several buffer sizes to allow examination of multiple spatial scales (e.g., Brauer et al. 2003, 2008; Jerrett et al. 2005). We used Census 2000 measures of population density (persons per square kilometer) and housing density (housing units per square kilometer) within each site’s census block group (U.S. Census Bureau 2002). We used monthly averages of daily high and low temperatures and precipitation collected at 31 weather stations (Western Regional Climate Center 2008). We used measurements at the nearest weather station; the average distance between each monitoring site and the nearest station was 6.8 km (range, 0.7–32 km). We used year and month terms to capture secular and seasonal variations in air pollutant concentrations.

### Model fitting procedures

Using a stepwise procedure previously described in detail (Rudra et al. 2010b), we fit multivariable linear regression models to quantify the relationship between each environmental characteristic and monthly average CO and PM_2.5_ concentrations. After determining the final exposure models, we measured the same environmental characteristics at each participant’s geocoded residential address reported during the interview. We used model coefficients and these measurements to predict participants’ monthly air pollutant exposures. We predicted exposures within each calendar month of pregnancy after approximating both the conception and delivery dates to the nearest calendar month, by assigning days 1–15 to the current month and rounding days 16–31 to the next month. We measured date of conception using maternal report of the date of the last menstrual period (LMP) and ultrasound at ≤ 20 weeks of gestation. LMP and ultrasound information were gathered by interview and medical record abstraction, respectively. If both LMP and ultrasound-based dates agreed within 14 days, we used the former. Among 4% of participants with dates differing by > 14 days, we used the ultrasound-based date.

The CO model included terms for year and month (indicators), street density within 500 m (quartile-based indicators), distance to the nearest major road (< 100, 101–1,000, > 1,000 m), and census tract population density (continuous). We previously reported that the model explained 73% of variation in CO concentrations (Rudra et al. 2010b). The root mean square error was 0.22 ppm (10% of the range of observed concentrations). The split-sample *R*^2^ was 0.71. CO concentrations were highest in winter, in the earliest years of the study, and in densely populated areas and were inversely related to the distance to the nearest major road. Concentrations were highest in areas with third-quartile street density.

The PM_2.5_ model included terms for year and month (indicators), street density within 250 m (tertile-based indicators), distance to the nearest major road (dichotomized at 2,000 m), and monthly averages of daily high temperatures and precipitation (quartile-based indicators). The model explained 47% of variance in regional PM_2.5_ concentrations. The split-sample *R*^2^ was 0.41, and the root mean square error was 2.5 μg/m^3^ (10% of the range). PM_2.5_ concentrations were highest in winter and in months with higher average temperature or precipitation; they were somewhat higher in earlier years but remained stable from 2003 onward. PM_2.5_ concentrations were inversely related to the distance to the nearest major road and, unexpectedly, to street density.

### Exposure estimation

For this analysis we defined *a priori* four exposure windows for each outcome of interest. For preeclampsia, exposure windows were periconceptional (the 7 months surrounding the month of conception), preconceptional (the average of the monthly concentrations in 3 months before pregnancy), postconceptional (the first 4 months of pregnancy, before preeclampsia can be diagnosed), and the peak monthly concentration in the 7-month periconceptional period (the month with the highest average concentration within that period). These windows were chosen to preclude exposures that may have occurred after diagnosis and to allow us to separately examine preconceptional exposures, which we hypothesized could increase maternal systemic oxidative stress or inflammation, and postconceptional exposures, which could adversely affect placentation (Brook 2008; Roberts et al. 2003). We chose a 7-month periconceptional window in order to have a roughly symmetric exposure period surrounding conception and to avoid exposures that could potentially occur after diagnosis. For preterm delivery, we examined average exposures within the first and second trimesters, the last 3 months of pregnancy, and the month of delivery. These windows were chosen to provide as direct comparisons as possible with the prior literature (Brauer et al. 2008; Liu et al. 2003; Parker et al. 2008; Ritz et al. 2007). The first- and second-trimester exposure windows were calculated based on the month of conception (months 1–3 and 4–6, respectively). The window based on the last 3 months of pregnancy was calculated by counting back from and including the month of delivery. A woman who conceived in April 1997 and delivered in December 1997 would have the following exposure windows: preconceptional, January–March; postconceptional, April–July; periconceptional, January–July; first trimester, April–June; second trimester, July–September; and third trimester, October–December 1997.

### Outcome measurements

We defined preeclampsia according to ACOG criteria using data obtained from medical records. The criteria are sustained pregnancy-induced hypertension (≥ 140/90 mmHg) and proteinuria (urine protein concentrations of 30 mg/dL or 1+ on two or more urine dipsticks) (ACOG 2002). We defined preterm delivery as a pregnancy lasting < 37 completed weeks of gestation. In secondary analyses, we examined length of gestation measured in days as an outcome, using the same exposure windows as in our preterm delivery analyses.

### Statistical analysis

We used multivariable logistic regression to model the relationships [odds ratios (ORs) and 95% confidence intervals (CIs)] between each pregnancy outcome and air pollutant exposure. Our alpha (type 1 error) level was 0.05. We used linear regression for models of length of gestation. For our primary analyses, we categorized air pollutant concentrations according to distribution quartiles. We also modeled air pollutant concentrations as continuous exposures. For these analyses, we report ORs associated with a 0.1-ppm increase in CO and a 0.5-μg/m^3^ increase in PM_2.5_. These gradients are roughly equivalent to the increments between deciles for both pollutants.

We evaluated the following characteristics, reported in interviews, as potential confounders: maternal age, nulliparity (no prior live births), prepregnancy body mass index (BMI), race/ethnicity, education, employment in early pregnancy, marital status, household income, smoking before and during pregnancy, usual hours/week of secondhand smoke exposure in and outside of the home in the year before pregnancy, regular recreational physical activity before and during pregnancy, and histories of asthma, diabetes, and chronic hypertension. We also evaluated year and month of conception as confounders because of temporal changes in both pollutant concentrations and preeclampsia risk (PSCAA 2007; Rudra and Williams 2005). *A priori*, we chose to include maternal age, race/ethnicity, prepregnancy BMI, and nulliparity as covariates because of their confounding effects in previous analyses of Omega Study data. Season and year were also included in multivariable models because of their influence on preeclampsia ORs. We found no evidence of confounding by other covariates using a 10% difference in magnitude between crude and adjusted ORs as the criterion for confounding. We found no evidence of poor model fit or influential observations after performing the following regression diagnostics: the Hosmer–Lemeshow test, comparison of observed and predicted outcomes within exposure and covariate strata, examination of Pearson and deviance residual plots, and examination of leverage plots (Hosmer and Lemeshow 2000).

We conducted several stratified analyses to evaluate the possibility of interaction by characteristics chosen *a priori* because of their strong relationships with either the outcomes or exposures. We stratified participants according to maternal age (< 35 or ≥ 35 years), parity (no vs. any prior live births), prepregnancy BMI (< 25 or ≥ 25 kg/m^2^), ever smoking or secondhand smoke exposure (neither vs. either), and employment in early pregnancy (used as a surrogate for time spent at home). We evaluated interaction by comparing ORs across strata; we defined interaction as a difference of > 20% in the magnitude of at least two of four category ORs. We also fitted models after excluding women with self-reported or medical record–indicated histories of prepregnancy diabetes or hypertension.

Finally, we conducted sensitivity analyses to examine the impact of extrapolation of the environmental characteristic predictors used in the air pollutant exposure models. For both CO and PM_2.5_, we classified women according to whether one or more values of the environmental characteristics used in the exposure estimation model were out of the range of values observed at the monitoring sites. For example, although the distance from each CO monitoring site to the nearest major road ranged from 0.2 to 1.3 km, the range of values at Omega participants’ addresses was much broader (0.01–92 km). Furthermore, a small proportion of women (≤ 4% for each exposure window) had one or more CO exposure estimates outside the range of the concentrations observed in the region. All predicted PM_2.5_ exposures fell within the range of observed concentrations. We hypothesized that our exposure estimates might be less accurate for women with one or more extrapolated values and that this inaccuracy might bias our estimates of association toward the null.

## Results

Median [interquartile range (IQR)] CO and PM_2.5_ concentrations observed at monitoring sites during the study period were 1.08 (0.80–1.38) ppm and 10.0 (8.3–12.4) μg/m^3^, respectively [Supplemental Material, Table 1 (doi:10.1289/ehp.1002947)]. Among study participants, median (IQR) predicted CO and PM_2.5_ exposures in the periconceptional period were 0.80 (0.59–1.02) ppm and 10.1 (8.7–11.4) μg/m^3^, respectively. By design, peak CO and PM_2.5_ exposures were higher [median (IQR): peak CO, 1.05 (0.85–1.27) ppm; peak PM_2.5_, 14.2 (12.6–15.1) μg/m^3^]. Distributions in the other six windows were similar to those in the periconceptional window: median CO concentrations ranged from 0.79 to 0.80 ppm, and median PM_2.5_ concentrations ranged from 9.5 to 12.6 μg/m^3^. The difference between distributions of observed CO concentrations and participants’ predicted exposures was due primarily to the fact that CO monitoring was more pervasive in the earlier years of the study (PSCAA 2007). CO and PM_2.5_ exposures were moderately correlated: Pairwise correlation coefficients ranged from 0.25 to 0.45 within exposure windows.

Table 1 shows distributions of selected participants’ characteristics and periconceptional CO and PM_2.5_ distributions within categories of those characteristics. Distributions for other exposure windows were similar. Participants were predominantly white, married, well educated, and nonsmokers. Predicted CO exposures were inversely associated with age, educational attainment, and household income and were higher among black or Hispanic women than among white women, unmarried women, and those who reported smoking or secondhand smoke exposure. Predicted CO exposures also declined sharply over the study period and were highest in women conceiving in winter. Although predicted PM_2.5_ exposures were generally less strongly related to maternal characteristics, they were also highest among black women, women with lower educational attainment or income, and those who conceived in winter.

A total of 117 women (3.3%) developed preeclampsia. Table 2 shows unadjusted and adjusted associations between periconceptional air pollutant exposures and preeclampsia. Because adjustment for year of conception strongly influenced the estimated associations, we show adjusted models without (model 2) and with (model 3) inclusion of year as a covariate. Fourth-quartile periconceptional CO concentrations were significantly associated with preeclampsia after adjustment for maternal characteristics and season of conception (fourth vs. first quartile: OR = 2.08; 95% CI, 1.16–3.72). However, this association became nonsignificant and inverted after further adjustment for year of conception (OR = 0.75; 95% CI, 0.42–1.64). Similarly, although each 0.1-ppm increase in CO was significantly associated with preeclampsia after adjustment for maternal characteristics and season of conception (OR = 1.07; 95% CI, 1.02–1.13), the association disappeared after further adjustment for year of conception (OR = 0.98; 95% CI, 0.91–1.06). The association between preeclampsia and periconceptional PM_2.5_ exposure was nonsignificant and weaker than for CO (fourth vs. first quartile OR, adjusted for maternal characteristics and season of conception, 1.63; 95% CI, 0.79–3.38). This association weakened somewhat after further adjustment for year of conception (OR = 1.41; 95% CI, 0.63–3.18). Unadjusted and adjusted associations with CO and PM_2.5_ in the preconceptional and postconceptional windows and the periconceptional month of peak exposure were similar to those in the periconceptional window (data not shown; tables of these and other secondary analyses are available from the authors upon request). For example, in models of each of these exposure windows, unadjusted for year of conception, ORs for each 0.1-ppm CO increase were all 1.07.

The incidence of preterm delivery was 10.5% (369 cases). Predicted air pollutant exposures in the last 3 months of pregnancy were not strongly or significantly associated with preterm delivery, and year of conception did not materially influence estimates of association (Table 3). For instance, fourth- versus first-quartile ORs were 0.88 (95% CI, 0.59–1.31) for CO exposure and 0.81 (95% CI, 0.49–1.34) for PM_2.5_ exposure in fully adjusted models. ORs for CO and PM_2.5_ exposures in the first and second trimesters and the last month of pregnancy were similar (data not shown). Multivariable linear regression analysis using gestational age at delivery as the outcome also produced similar results: We found no statistically significant or strong associations with exposures to either air pollutant (data not shown).

Analyses of models stratified by maternal age, parity, prepregnancy BMI, employment, and smoking or secondhand smoke exposure did not provide evidence that any of these characteristics modified associations between air pollutant exposures and either outcome (data not shown). CO–preeclampsia associations were robust to exclusion of women with prepregnancy diabetes (*n* = 105) or hypertension (*n* = 115; data not shown). We examined the sensitivity of our results to participants’ residential values of environmental characteristics used as predictors in our air pollutant exposure models. A total of 1,930 women (55%) resided in an area at which one or more CO model predictors were outside the range observed at monitoring sites. Proportions for individual predictors were 7.9% for population density, 9.2% for street density, and 47.3% for distance to the nearest major road (only 0.1% lived < 0.2 km from a major road; 47.2% lived > 1.3 km). For 904 participants (25.8%), the street density measure used in the PM_2.5_ model was higher (24.2%) or lower (1.6%) than the range observed at monitoring sites. Associations between preeclampsia and air pollutant exposures were slightly stronger in the subset of women with no out-of-range model predictors. For example, the OR for a 0.1-ppm increase in periconceptional CO exposure was 1.10 (95% CI, 0.98–1.23) among 1,579 women with no out-of-range model predictors and 1.06 (95% CI, 0.99–1.12) among 1,930 women with one or more out-of-range predictors (ORs were adjusted for maternal characteristics and season but not year). Associations with preterm delivery also were similar between the two subgroups (data not shown). Finally, exclusion of the small proportion of women with predicted CO exposures outside the range of concentrations observed in the area (between 44 and 101 observations for each of the CO exposure windows) did not materially affect our results (data not shown).

## Discussion

We found little evidence of strong associations between either predicted CO or PM_2.5_ exposure and preterm delivery risk in this setting. Although point estimates suggested increased risk of preeclampsia associated with PM_2.5_ exposure, CIs were wide. We found statistically significant, positive associations between preeclampsia and CO in all examined exposure windows. However, these associations disappeared after adjustment for year of conception.

We debated whether adjustment for year of conception was appropriate when examining the association between CO exposure and preeclampsia. CO concentrations in the four counties studied here declined 70 ppb/year (95% CI, 63–77 ppb/year), on average, between 1996 and 2006. Regional concentrations of PM_2.5_ and other criteria air pollutants remained stable or declined much less markedly (PSCAA 2007). The incidence of preeclampsia in our cohort declined over time independently of changes in maternal age, race/ethnicity, parity, prepregnancy BMI, and smoking history (adjusted decline in incidence, 5.3 cases/1,000 per year; 95% CI, 3.0–7.6/1,000 per year). No other covariate reported in Table 1 explained the secular decrease in incidence (data not shown). Because we consistently applied ACOG criteria to medical record information rather than relying on physician diagnosis, changes in diagnostic criteria cannot explain the decline in incidence. If regional CO declines were causally related to declines in preeclampsia incidence, adjustment for year of conception would be inappropriate. However, we cannot discount the possibility that secular change in some other risk factor may explain the decline in preeclampsia incidence. Adjustment for calendar year showed that differences in CO exposures among participants who conceived in the same year were not associated with preeclampsia risk. However, year of conception and CO exposures were strongly correlated in this population. More than half (52%) of participants with fourth-quartile periconceptional CO exposure conceived in the first 3 years of the study, whereas 43% of those with first-quartile exposure conceived in the last 2 years. As previously described in detail, the CO model captured the fact that most CO variation in this setting was temporal rather than spatial (Rudra et al. 2010b). The lesser spatial CO variability limited our ability to examine spatial contrasts and may also explain the lack of association after adjustment for year of conception.

There have been few studies of preeclampsia in relation to air pollutant exposures. We previously reported suggestive but nonsignificant associations between CO and preeclampsia among the subset of 1,699 Omega Study participants who enrolled between 1996 and 2002 (Rudra and Williams 2006). The third- versus first-tertile periconceptional CO-adjusted OR was 1.73 (95% CI, 0.91–3.27), and PM_2.5_ exposures were not strongly related to preeclampsia. Similarly, Woodruff et al. (2008) reported positive associations between nearest-monitor CO concentrations and preeclampsia in an analysis of approximately 2.3 million California residents delivering between 1996 and 2004 (fourth vs. first quartile: adjusted OR = 1.08; 95% CI, 1.02–1.13; quartile cut-points not reported). They adjusted this estimate of association for year, season, and other characteristics. They did not observe associations with PM_2.5_ exposures. Van den Hooven et al. (2009) observed no association between residential proximity to traffic (a proxy of air pollutant exposure) and preeclampsia risk in 7,339 Rotterdam residents enrolled in a population-based cohort. Most recently, Wu et al. (2009) reported that traffic-generated PM_2.5_ exposures predicted by dispersion models were significantly associated with increased preeclampsia risk [fourth vs. first quartile: adjusted OR = 1.42; 95% CI, 1.26–1.59; mean (IQR) PM_2.5_ exposures, 1.82 (1.35) μg/m^3^]. Traffic-generated nitrogen oxides were similarly associated (fourth vs. first quartile: adjusted OR = 1.33; 95% CI, 1.18–1.48). They did not examine CO exposures.

Given the similarities between preeclampsia and atherosclerotic cardiovascular disease (Kaaja and Greer 2005), many of the hypothesized mechanisms by which air pollutants increase cardiovascular risk such as inflammation, oxidative stress, and endothelial dysfunction (Brook 2008) may also apply to preeclampsia. Furthermore, hypoxia at the fetal–maternal interface secondary to impaired placentation has been hypothesized to cause dissemination of free radicals that trigger preeclampsia in susceptible women (Roberts et al. 2003). Inhaled CO may also induce hypoxia because it displaces oxygen from hemoglobin, forming carboxyhemoglobin (Scherer 2006). Even fairly low maternal carboxyhemoglobin concentrations can reduce oxygen transfer across the placenta (Hsia 1998). We previously reported that maternal blood carboxyhemoglobin in early pregnancy was associated with preeclampsia risk among Omega participants, although only within the subgroup of women with prior live births (> 1% vs. 0.7% carboxyhemoglobin: adjusted OR = 4.1; 95% CI, 1.3–12.9; parity interaction *p*-value = 0.01) (Rudra et al. 2010a). In contrast, the association we observed in the present study between CO and preeclampsia exposure was not modified by parity.

The literature on CO, PM_2.5_, and preterm delivery provides mixed results and no strong evidence of specific exposure windows (Brauer et al. 2008; Darrow et al. 2009; Liu et al. 2003; Parker et al. 2008; Ritz et al. 2007; Wu et al. 2009; Zeka et al. 2008). Two studies of the topic were conducted in Vancouver, British Columbia, an area with an airshed similar to that of the Puget Sound region (Brauer et al. 2008; Liu et al. 2003). Among about 230,000 women delivering between 1987 and 1998, Liu et al. (2003) observed increased preterm delivery associated with each 1.0-ppm increase in CO exposure in the last month of pregnancy (adjusted OR = 1.08; 95% CI, 1.01–1.15) but not the first month (adjusted OR = 0.95; 95% CI, 0.89–1.01).They estimated CO exposure as the average within the study region and did not examine PM_2.5_. Brauer et al. (2008) used inverse-distance weighting to predict CO and PM_2.5_ exposures for approximately 70,000 women delivering between 1999 and 2002. Each 1-μg/m^3^ increase in PM_2.5_ exposure over the entire pregnancy was associated with a 6% increase in the odds of preterm delivery (OR = 1.06; 95% CI, 1.01–1.11). CO exposure was not associated with delivery at < 37 weeks of gestation but was associated with delivery at < 30 weeks of gestation [adjusted OR, per 100-μg/m^3^ (~ 0.09 ppm) increase, 1.16; 95% CI, 1.01–1.33]. They found no exposure windows of greater or lesser relevance.

We used prediction models containing month, year, and land use terms to capture both spatial and temporal variations in CO and PM_2.5_ exposures. A strength of these models was the opportunity they provided to examine multiple exposure windows for both outcomes. As measured by *R*^2^ statistics, these models performed similarly to others described in the literature (Brauer et al. 2003; Clougherty et al. 2008; Henderson et al. 2007; Moore et al. 2007). We relied on local air monitoring data in designing our models rather than collecting data for the purposes of this study. Although this approach is cost-effective and convenient, the monitoring sites may not have been optimally located for the purposes of individual exposure prediction. CO sites, particularly, were often closer to major roads or in denser areas than were many participants’ residences. However, our results were robust to exclusion of women who lived in areas that differed substantially from monitoring sites with respect to model predictors.

We previously reported that our CO model–based exposure estimates were moderately correlated with contemporaneously measured whole-blood carboxyhemoglobin concentrations in this study population (Rudra et al. 2010b). There is no known biomarker of PM_2.5_ exposure, and because of budgetary constraints we were unable to validate our PM_2.5_ model against independently collected air quality measurements. The *R*^2^ for the PM_2.5_ model was lower than that for the CO model, primarily because year, traffic, and street density measures were less strongly predictive of PM_2.5_ than of CO. Research has shown that errors in air pollutant exposure estimates such as those used here have both Berkson-like and classical-like components, and that it is theoretically possible that these errors are biased away from the null (Szpiro et al. 2010). However, simulation studies using data from a network of a small number of monitors have found bias toward the null and wider CIs (Kim et al. 2009). Thus, we believe that limitations of our estimation method may have resulted in misclassification of PM_2.5_ and CO exposures toward the null. The misclassification of PM_2.5_ exposures may have masked associations with preeclampsia and preterm delivery. Errors and uncertainties in both CO and PM_2.5_ models may have also arisen from imprecision in geocoding, a limited number of locations (none of which were participants’ locations), rounding of exposure windows to the nearest month (which also hampered our ability to differentiate among exposure windows), and inaccurate land use measures. Because we based exposures on self-reported residential address, errors may have also arisen if participants moved soon before or after the interview; longer exposure windows may have been more greatly affected by this source of misclassification. Although employment status, used as a rough surrogate for time spent at home, did not influence our findings, we were not able to capture exposures experienced at other locations and during commuting. Additionally, our models did not capture exposures arising from indoor sources, although we were able to evaluate self-reported secondhand smoke exposure as a potential confounder. If missing nonambient exposures were unrelated to ambient exposures, they are unlikely to have affected our estimates of association (Sheppard et al. 2005).

Most previous studies of air pollution and pregnancy outcomes have been based on birth registries or hospital databases (Brauer et al. 2008; Darrow et al. 2009; Liu et al. 2003; Parker et al. 2008; Ritz et al. 2007; Wilhelm and Ritz 2005; Wu et al. 2009; Zeka et al. 2008), with at least two exceptions (Ritz et al. 2007; van den Hooven et al. 2009). Our ability to rely on data from an epidemiologic cohort was a significant strength, because we were able to evaluate many characteristics that would not have been available from birth records as potential confounders and effect modifiers. Furthermore, abstraction of medical record data to determine outcomes strengthened our study by precluding limitations arising from inaccuracies in gestational age and underreporting of preeclampsia in birth records (Lydon-Rochelle et al. 2005; Martin 2007). However, reliance on cohort data resulted in fewer outcomes and less precise estimates than we would have been able to obtain with a larger regional registry. Because of limited medical record data on preeclampsia severity, we could not evaluate the relationships between preeclampsia severity or gestational age at onset and air pollutant exposures. However, in post hoc analyses we observed that associations were similar between preeclampsia associated with term delivery (*n* = 72) and preeclampsia associated with preterm delivery (*n* = 45; data not shown). The Omega Study’s high participation and retention rates reduced the likelihood that selection bias and missing data influenced our estimates of association. Study participants had higher income and educational attainment, on average, than did Washington State pregnant women (Rudra and Williams 2005). Participants were also more likely to be white and non-Hispanic. Therefore, these results may not be generalizable to other demographic groups, particularly those with higher risks of these outcomes.

Although CO concentrations were quite high at the start of the study period (the region attained U.S. EPA compliance in 1996), they declined rapidly. PM_2.5_ concentrations were moderate compared with other U.S. cities; most of the region was in compliance with the current U.S. EPA standard throughout the study period (PSCAA 2007). These results may not be generalizable to women living in areas with markedly different air pollutant concentrations. Future studies of these outcomes in high-pollutant settings would be useful contributions to the literature.

## Conclusions

We found strong positive associations between CO and preeclampsia risk. Because both factors declined during the study period, we cannot exclude the possibility that secular changes in a preeclampsia risk factor unidentified in this study may explain the association. Our results do not provide evidence that PM_2.5_ and CO strongly influence risks of preterm delivery and preeclampsia among western Washington State women.

## Figures and Tables

**Table 1:** Distributions of selected characteristics and estimated periconceptional CO and PM_2.5_ exposures: Seattle and Tacoma, Washington, 1996–2006 (*n* = 3,509).

		Median (IQR)	
Characteristic	*n* (%)	CO (ppm)	PM_2.5_ (μg/m^3^)
Age (years)			
≤ 20	39 (1.1)	0.94 (0.83–1.22)	10.2 (8.7–11.8)
21–34	2,293 (66.4)	0.82 (0.61–1.04)	10.1 (8.7–11.4)
35–39	966 (27.5)	0.75 (0.54–0.96)	10.2 (9.0–11.5)
≥ 40	211 (6.0)	0.77 (0.55–1.02)	10.0 (8.6–11.2)

Parity			
Nulliparous	2,087 (59.5)	0.85 (0.64–1.08)	10.1 (8.7–11.4)
Parous	1,422 (40.5)	0.73 (0.55–0.94)	10.2 (8.7–11.5)

Prepregnancy BMI (kg/m^2^)			
< 18.5	146 (4.2)	0.80 (0.60–1.02)	10.2 (9.0–11.6)
18.5–24.9	2,455 (69.9)	0.81 (0.59–1.02)	10.1 (8.7–11.4)
25.0–29.9	586 (16.7)	0.80 (0.57–1.01)	10.1 (8.7–11.5)
≥ 30.0	322 (9.2)	0.81 (0.60–1.09)	10.2 (8.8–11.5)

Race/ethnicity			
Non-Hispanic white	3,012 (85.8)	0.79 (0.59–1.02)	10.1 (8.7–11.5)
Non-Hispanic black	72 (2.1)	0.91 (0.73–1.11)	10.7 (8.8–11.8)
Hispanic	63 (1.8)	0.97 (0.78–1.10)	10.2 (8.8–11.0)
Asian/Pacific Islander	262 (7.5)	0.82 (0.59–1.01)	10.2 (9.0–11.4)
Other	100 (2.8)	0.76 (0.56–1.01)	9.8 (8.6–11.1)

Education^a^			
High school	126 (3.8)	0.85 (0.67–1.10)	10.5 (6.9–11.7)
Vocational or some college	494 (14.9)	0.86 (0.63–1.10)	10.2 (8.8–11.6)
Completed college	1,594 (47.9)	0.79 (0.60–1.01)	10.2 (8.8–11.3)
Postgraduate	1,115 (33.5)	0.79 (0.58–1.02)	10.0 (8.6–11.3)

Employed during pregnancy^a^			
Yes	2,717 (82.0)	0.82 (0.61–1.05)	10.1 (8.7–11.4)
No	599 (18.0)	0.78 (0.57–0.97)	10.3 (8.8–11.6)

Marital status^a^			
Married/living as married	3,295 (93.9)	0.79 (0.59–1.01)	10.1 (8.7–11.4)
Unmarried	214 (6.1)	0.89 (0.67–1.19)	10.3 (8.6–11.6)

Annual household income (US$)^a^			
< 30,000	102 (3.2)	0.98 (0.77–1.21)	10.4 (8.6–11.6)
31,000–69,999	645 (20.0)	0.92 (0.69–1.13)	10.2 (8.7–11.4)
≥ 70,000	2,473 (76.8)	0.77 (0.58–0.99)	10.1 (8.9–11.4)

Smoking status^a^			
Never	2,394 (72.1)	0.81 (0.60–1.02)	10.1 (8.7–11.4)
Before pregnancy	732 (22.1)	0.78 (0.60–1.05)	10.2 (8.7–11.5)
Before and during pregnancy	194 (5.8)	0.88 (0.62–1.12)	10.3 (8.8–11.3)

Usual hours/week of secondhand smoke exposure in the year before pregnancy			
None	2,491 (71.0)	0.76 (0.56–0.96)	10.2 (8.8–11.5)
< 7	918 (26.2)	0.93 (0.70–1.13)	10.0 (8.6–11.4)
≥ 7	100 (2.8)	1.06 (0.88–1.25)	10.7 (9.4–11.7)

Year of conception			
1996–1999	745 (21.2)	1.10 (0.93–1.28)	9.9 (8.3–11.4)
2000–2002	1,214 (34.6)	0.85 (0.68–1.04)	10.2 (8.6–11.3)
2003–2004	821 (23.4)	0.66 (0.50–0.83)	10.5 (9.2–11.8)
2005–2006	729 (20.8)	0.59 (0.40–0.76)	9.9 (8.8–11.4)

Season of conception			
Winter (Dec–Feb)	859 (24.5)	0.91 (0.72–1.12)	11.7 (11.2–12.5)
Spring (Mar–May)	878 (25.0)	0.72 (0.51–0.95)	9.3 (8.5–10.1)
Summer (June–Aug)	923 (26.3)	0.71 (0.48–0.89)	8.4 (7.7–9.3)
Autumn (Sept–Nov)	849 (24.2)	0.88 (0.67–1.08)	10.9 (10.1–11.7)

a Numbers in subgroups do not equal overall number because of missing data.

**Table 2:** Associations between periconceptional air pollutant exposures^a^ and preeclampsia.

		OR (95% CI)		
Exposure	*n* (cases/total)	Unadjusted model 1	Adjusted model 2^b^	Adjusted model 3^c^
Periconceptional CO				
Categorized by quartiles (ppm)				
0.09–0.59	21/878	1.00 (Referent)	1.00 (Referent)	1.00 (Referent)
0.60–0.80	27/877	1.30 (0.73–2.31)	1.44 (0.79–2.62)	1.04 (0.55–1.96)
0.81–1.02	30/877	1.45 (0.82–2.54)	1.65 (0.91–3.00)	0.86 (0.42–1.75)
1.03–3.73	39/877	1.90 (1.11–3.26)	2.08 (1.16–3.72)	0.75 (0.42–1.64)
Per 0.1-ppm increase		1.07 (1.02–1.12)	1.07 (1.02–1.13)	0.98 (0.91–1.06)

Periconceptional PM_2.5_				
Categorized by quartiles (μg/m^3^)				
5.9–8.7	31/878	1.00 (Referent)	1.00 (Referent)	1.00 (Referent)
8.8–10.1	24/877	0.77 (0.45–1.32)	0.79 (0.45–1.39)	0.78 (0.43–1.41)
10.2–11.4	32/882	1.03 (0.62–1.70)	1.50 (0.80–2.81)	1.38 (0.71–2.70)
11.5–15.1	30/872	0.97 (0.58–1.62)	1.63 (0.79–3.38)	1.41 (0.63–3.18)
Per 0.5-μg/m^3^ increase		1.00 (0.94–1.05)	1.04 (0.95–1.12)	1.02 (0.93–1.12)

a Average of the estimated monthly average concentrations in the 7 calendar months surrounding conception.

b Model 2 was adjusted for age (< 20, 21–35, ≥ 35 years), race/ethnicity (white, black, Hispanic, other), nulliparity (yes/no), prepregnancy BMI (< 20, 20–24.9, 25–29.9, ≥ 30 kg/m^2^), smoking history (never, before pregnancy only, during early pregnancy), and season of conception.

c Model 3 was adjusted for model 2 covariates and year of conception (indicator terms).

**Table 3:** Associations between air pollutant exposures in the last 3 months of pregnancy and preterm delivery.

		OR (95% CI)		
Exposure	*n* (cases/total)	Unadjusted model 1	Adjusted model 2^a^	Adjusted model 3^b^
CO in the last 3 months of pregnancy				
Categorized by quartiles (ppm)				
0.03–0.57	87/878	1.00 (Referent)	1.00 (Referent)	1.00 (Referent)
0.58–0.79	92/877	1.07 (0.78–1.45)	1.00 (0.73–1.38)	0.99 (0.72–1.37)
0.80–1.04	100/877	1.17 (0.86–1.59)	1.08 (0.78–1.49)	1.08 (0.77–1.53)
1.05–3.77	90/877	1.04 (0.76–1.42)	0.89 (0.63–1.26)	0.88 (0.59–1.31)
Per 0.1-ppm increase		1.00 (0.97–1.03)	0.98 (0.94–1.01)	0.97 (0.93–1.01)

PM_2.5_ in the last 3 months of pregnancy				
Categorized by quartiles (μg/m^3^)				
5.2–7.9	80/878	1.00 (Referent)	1.00 (Referent)	1.00 (Referent)
8.0–9.9	94/877	1.01 (0.88–1.65)	1.19 (0.85–1.66)	1.21 (0.86–1.70)
10.0–12.6	102/882	1.32 (0.97–1.80)	1.14 (0.77–1.68)	1.14 (0.76–1.70)
12.7–17.2	93/872	1.19 (0.87–1.63)	0.83 (0.52–1.34)	0.81 (0.49–1.34)
Per 0.5-μg/m^3^ increase		1.01 (0.99–1.03)	0.99 (0.96–1.02)	0.97 (0.91–1.04)

a Model 2 was adjusted for age (< 20, 21–35, ≥ 35 years), race/ethnicity (white, black, Hispanic, other), nulliparity (yes/no), prepregnancy BMI (< 20, 20–24.9, 25–29.9, ≥ 30 kg/m^2^), smoking history (never, before pregnancy only, during early pregnancy), and season of conception.

b Model 3 was adjusted for model 2 covariates and year of conception (indicator terms).

## References

[b1-ehp-119-886] ACOG (American College of Obstetricians and Gynecologists), Committee on Practice Bulletins—Obstetrics (2002). Diagnosis and management of preeclampsia and eclampsia. ACOG practice bulletin 33. Obstet Gynecol.

[b2-ehp-119-886] Ananth CV, Savitz DA, Luther ER, Bowes WA (1997). Preeclampsia and preterm birth subtypes in Nova Scotia, 1986 to 1992. Am J Perinatol.

[b3-ehp-119-886] Brauer M, Hoek G, van Vliet P, Meliefste K, Fischer P, Gehring U (2003). Estimating long-term average particulate air pollution concentrations: application of traffic indicators and geographic information systems. Epidemiology.

[b4-ehp-119-886] Brauer M, Lencar C, Tamburic L, Koehoorn M, Demers P, Karr C (2008). A cohort study of traffic-related air pollution impacts on birth outcomes. Environ Health Perspect.

[b5-ehp-119-886] Brook RD (2008). Cardiovascular effects of air pollution. Clin Sci (Lond).

[b6-ehp-119-886] Butler CL, Williams MA, Sorensen TK, Frederick IO, Leisenring WM (2004). Relation between maternal recreational physical activity and plasma lipids in early pregnancy. Am J Epidemiol.

[b7-ehp-119-886] Clougherty JE, Wright RJ, Baxter LK, Levy JI (2008). Land use regression modeling of intra-urban residential variability in multiple traffic-related air pollutants. Environ Health.

[b8-ehp-119-886] Darrow LA, Klein M, Flanders WD, Waller LA, Correa A, Marcus M (2009). Ambient air pollution and preterm birth: a time-series analysis. Epidemiology.

[b9-ehp-119-886] Eder W, Ege MJ, von Mutius E (2009). The asthma epidemic. N Engl J Med.

[b10-ehp-119-886] Hamilton BE, Martin JA, Ventura SJ (2006). Births: preliminary data for 2008. Natl Vital Stat Rep.

[b11-ehp-119-886] Henderson SB, Beckerman B, Jerrett M, Brauer M (2007). Application of land use regression to estimate long-term concentrations of traffic-related nitrogen oxides and fine particulate matter. Environ Sci Technol.

[b12-ehp-119-886] Hosmer DW, Lemeshow S (2000). Applied Logistic Regression.

[b13-ehp-119-886] Hsia CC (1998). Respiratory function of hemoglobin. N Engl J Med.

[b14-ehp-119-886] IOM (Institute of Medicine, Committee on Understanding Premature Birth and Assuring Healthy Outcomes) (2007). Preterm Birth: Causes, Consequences, and Prevention.

[b15-ehp-119-886] Jerrett M, Arain A, Kanaroglou P, Beckerman B, Potoglou D, Sahsuvaroglu T (2005). A review and evaluation of intraurban air pollution exposure models. J Expo Anal Envrion Epidemiol.

[b16-ehp-119-886] Kaaja RJ, Greer IA (2005). Manifestations of chronic disease during pregnancy. JAMA.

[b17-ehp-119-886] Kannan S, Misra DP, Dvonch JT, Krishnakumar A (2006). Exposures to airborne particulate matter and adverse perinatal outcomes: a biologically plausible mechanistic framework for exploring potential effect modification by nutrition. Environ Health Perspect.

[b18-ehp-119-886] Kim SY, Sheppard L, Kim H (2009). Health effects of long-term air pollution: influence of exposure prediction methods. Epidemiology.

[b19-ehp-119-886] Liu S, Krewski D, Shi Y, Chen Y, Burnett RT (2003). Association between gaseous ambient air pollutants and adverse pregnancy outcomes in Vancouver, Canada. Environ Health Perspect.

[b20-ehp-119-886] Lydon-Rochelle MT, Holt VL, Cardenas V, Nelson JC, Easterling TR, Gardella C (2005). The reporting of pre-existing maternal medical conditions and complications of pregnancy on birth certificates and in hospital discharge data. Am J Obstet Gynecol.

[b21-ehp-119-886] Martin JA (2007). United States vital statistics and the measurement of gestational age. Paediatr Perinat Epidemiol.

[b22-ehp-119-886] Moore DK, Jerrett M, Mack WJ, Kunzli N (2007). A land use regression model for predicting ambient fine particulate matter across Los Angeles, CA. J Environ Monit.

[b23-ehp-119-886] Newstead J, von Dadelszen P, Magee LA (2007). Preeclampsia and future cardiovascular risk. Expert Rev Cardiovasc Ther.

[b24-ehp-119-886] Parker JD, Mendola P, Woodruff TJ (2008). Preterm birth after the Utah Valley Steel Mill closure: a natural experiment. Epidemiology.

[b25-ehp-119-886] PSCAA (Puget Sound Clean Air Agency) (2007). 2006 Air Quality Data Summary.

[b26-ehp-119-886] Ritz B, Wilhelm M, Hoggatt KJ, Ghosh JK (2007). Ambient air pollution and preterm birth in the Environment and Pregnancy Outcomes Study at the University of California, Los Angeles. Am J Epidemiol.

[b27-ehp-119-886] Roberts JM, Pearson GD, Cutler JA, Lindheimer MD (2003). Summary of the NHLBI Working Group on Research on Hypertension during Pregnancy. Hypertens Pregnancy.

[b28-ehp-119-886] Rudra C, WIlliams M (2005). Monthly variation in preeclampsia prevalence: Washington State, 1987–2001. J Mat Fet Neonat Med.

[b29-ehp-119-886] Rudra C, Williams M (2006). A prospective study of periconceptional ambient air pollutant exposures and preeclampsia risk [Abstract]. Epidemiology.

[b30-ehp-119-886] Rudra CB, Williams MA, Schiff MA, Koenig JQ, Dills R, Yu J (2010a). A prospective study of maternal carboxyhaemoglobin and pre-eclampsia risk. Paediatr Perinat Epidemiol.

[b31-ehp-119-886] Rudra CB, Williams MA, Sheppard L, Koenig JQ, Schiff MA, Frederick IO (2010b). Relation of whole blood carboxyhemoglobin concentration to ambient carbon monoxide exposure estimated using regression. Am J Epidemiol.

[b32-ehp-119-886] Scherer G (2006). Carboxyhemoglobin and thiocyanate as biomarkers of exposure to carbon monoxide and hydrogen cyanide in tobacco smoke. Exp Toxicol Pathol.

[b33-ehp-119-886] Sheppard L, Slaughter C, Schildcrout J, Liu L-JS, Lumley T (2005). Exposure and measurement contributions to estimates of acute air pollution effects. J Expo Anal Environ Epidemiol.

[b34-ehp-119-886] Szpiro AA, Sheppard L, Lumley T (2010). Efficient Measurement Error Correction with Spatially Misaligned Data. UW Biostats Working Paper Series. Working Paper 350.

[b35-ehp-119-886] U.S. Census Bureau (2002). 108th Congressional District Census 2000 Summary File 1.

[b36-ehp-119-886] van den Hooven EH, Jaddoe VW, de Kluizenaar Y, Hofman A, Mackenbach JP, Steegers EA (2009). Residential traffic exposure and pregnancy-related outcomes: a prospective birth cohort study. Environ Health.

[b37-ehp-119-886] Washington State Department of Transportation (2002). 2001 Annual Traffic Report: Revision #1.

[b38-ehp-119-886] Western Regional Climate Center (2008). Western U.S. Climate Historical Summaries: Washington Climate Summaries.

[b39-ehp-119-886] Wilhelm M, Ritz B (2005). Local variations in CO and particulate air pollution and adverse birth outcomes in Los Angeles County, California, USA. Environ Health Perspect.

[b40-ehp-119-886] Woodruff T, Morello-Frosch R, Jesdale B (2008). Air pollution and preeclampsia among pregnant women in California, 1996–2004 [Abstract]. Epidemiology.

[b41-ehp-119-886] Wu J, Ren C, Delfino RJ, Chung JH, Wilhelm M, Ritz B (2009). Association between local traffic-generated air pollution and preeclampsia and preterm delivery in the South Coast Air Basin of California. Environ Health Perspect.

[b42-ehp-119-886] Zeka A, Melly SJ, Schwartz J (2008). The effects of socioeconomic status and indices of physical environment on reduced birth weight and preterm births in eastern Massachusetts. Environ Health.

